# EGR-1/ASPP1 inter-regulatory loop promotes apoptosis by inhibiting cyto-protective autophagy

**DOI:** 10.1038/cddis.2017.268

**Published:** 2017-06-08

**Authors:** Kunming Zhao, Miao Yu, Yifu Zhu, Dong Liu, Qiong Wu, Ying Hu

**Affiliations:** 1 School of Life Science and Technology, Harbin Institute of Technology, Harbin, China; 2 School of Chemical Engineering and Technology, Harbin Institute of Technology, Harbin, China; 3 Shenzhen Graduate School of Harbin Institute of Technology, Harbin, China

## Abstract

The decrease of ASPP1 (Apoptosis-Stimulating Protein of p53 1), a known p53 activator, has been linked to carcinogenesis and the cytotoxic resistance in various cancers, yet the underlying mechanisms of ASPP1 expression and its complex functions are not yet clear. Here, we report that ASPP1 forms an inter-regulatory loop with Early Growth Response 1 (EGR-1), and promotes apoptosis via inhibiting cyto-protective autophagy, independent of the well-documented p53-dependent mechanisms. We show that ASPP1 mRNA and protein were remarkably elevated by ectopic EGR-1 expression or endogenous EGR-1 activation, in cells with different tissue origins and p53 status. Conversely, RNAi-mediated EGR-1 knockdown suppressed ASPP1. The further mechanism studies revealed that ASPP1 promoter, mapped to −283/+88, which contained three conserved EGR-1 binding sites, was required for both binding and transactivity of EGR-1. In addition, we demonstrate that ASPP1 promoted EGR-1 in a positive feedback loop by preventing proteasome-mediated EGR-1 degradation or promoting EGR-1 nuclear import in response to anticancer natural compound Quercetin. Furthermore, albeit activating p53 in the nucleus is the well-studied function of ASPP1, we found that ASPP1 was predominately localized in the cytoplasm. Interestingly, the cytoplasmic ASPP1 retained its pro-apoptosis capability. Mechanistically, ASPP1 suppressed Atg5–Atg12 and also bound with Atg5–Atg12 to prevent its further complex formation with Atg16, resulting in the inhibition of cyto-protective autophagy. In conclusion, our results provide new insights into EGR-1/ASPP1 regulatory loop in sensitizing Quercetin-induced apoptosis. EGR-1/ASPP1, therefore, may be potentially used as therapeutic targets to improve cancer’s response to pro-apoptosis treatments.

Apoptosis-Stimulating Protein of p53 1 (ASPP1), together with ASPP2 and iASPP (inhibitor of ASPP), constitutes ASPP family. All family proteins are characterized by the highly conserved C-terminal structures, including ankyrin repeats, an SH3 domain, and a proline-rich region, by which ASPPs can directly interact with p53, and selectively regulate the transcription activity of p53 toward pro-apoptosis targets. ASPP1 and ASPP2 promote apoptosis, whereas iASPP inhibits it.^1, 2^ Because evasion of apoptosis is a hallmark of cancer, it comes as no surprise that apoptosis enhancer ASPP1 has been frequently observed to decrease at mRNA and/or protein levels in human cancers.^3, 4, 5^ So far, the anticancer activity of ASPP1 is largely dependent on p53, the precise nuclear localization is thus critical. However, ASPP1 is frequently detected in the cytoplasm,^6^ suggesting that unknown suppressor function of cytoplasmic ASPP1 may also exist. Interestingly, the tumor-suppressive function of ASPP1 has been recently demonstrated in a transgenic mouse study, showing that this function of ASPP1 in preventing the occurrence of hematological malignancy is both p53-dependent and -independent.^7, 8^ Although the p53-dependent mechanisms have been well demonstrated in the literature,^9, 10^ p53-independent mechanisms are yet kept largely unknown.

It is also noteworthy that loss of ASPP1 is correlated with drug resistance.^1^ Recent studies have shown that anticancer agents can simultaneously promote apoptosis and autophagy. Paradoxically, the induced autophagy is often related to the elevated resistance to apoptosis. It remains an important issue to understand the underlying mechanisms for the conversion between apoptosis and autophagy, which may be decisive for cancer cell fates upon treatments. Interestingly, it has been shown that ASPP family member ASPP2 influences pancreatic or colorectal cancer cells’ responses to chemotherapy-induced cell death by inhibiting autophagy.^11, 12^ ASPP2 has been reported to promote autophagy in liver cancers, which leads to autophagic cell death.^13^ ASPP1 is also essential in cell fates determination in response to cellular stresses.^4, 14^ However, whether it is involved in the regulation of the conversion between autophagy and apoptosis yet remains unknown.

In addition to the identification of its novel downstream functions, unveiling the mechanisms underlying ASPP1 regulation is also important, because both may offer new insight into the cancer treatment strategies by exploiting apoptosis. Yet, the only reported transcription factor that contributes to the regulation of ASPP1 is E2F family. Previous studies show that ASPP1 can be elevated at transcription level by E2F1 in response to DNA damage-induced apoptosis.^15, 16^ It remains to be determined whether other factors may also exist in regulating ASPP1 transcription.

The transcription factor EGR-1 (Early growth response protein 1), also known as *NGFI-A, TIS8, Krox-24* and *Zif268*, is a zinc-figure nuclear transcription factor and can be rapidly and transiently induced by various stimuli, such as H_2_O_2_, ionizing radiation, ultraviolet light (UV), hypoxia, serum and growth factors.^17, 18^ On activation, it binds to the GC-rich consensus sequences on gene promoters and regulates the downstream target transcription, leading to various biological effects, such as apoptosis, proliferation, angiogenesis, migration and differentiation, in a cellular context and stimulus-dependent manner.^19, 20^ For instance, elevated EGR-1 promotes cell proliferation and may contribute to the occurrence of prostate cancers.^18^ On the contrary, numbers of other studies have suggested that EGR-1 is a tumor suppressor. First of all, EGR-1 was dramatically reduced in human malignancies, such as breast cancer,^21^ non-small cell lung cancer,^22^ glioma and acute myeloid leukemias.^23^ In addition, EGR-1 has been demonstrated as a haploinsufficient tumor suppressor in leukemogenesis and has important roles in maintaining hematopoietic stem cell (HSC) quiescence in transgenic mice models.^24, 25^ Furthermore, EGR-1 null mice are prone to skin cancers in a two-step skin carcinogenesis study.^26^ In support of the anticancer functions of EGR-1 described above, a number of tumor-suppressor genes, such as p53 and its family member p73,^27^ PTEN,^28^ transforming growth factor-*β*1,^29^ p21,^30^ are identified as direct transcriptional targets of EGR-1. Furthermore, EGR-1 is also subjected to the regulation of its targets, p53, p73 and EGR-1 itself, in a positive feedback loop at transcriptional levels. More complicatedly, p53-independent pro-apoptosis functions of EGR-1 have been also reported.^31, 32^ No doubts, dissecting the downstream targets will facilitate the understanding of complex network of EGR-1 signaling.

In the present study, we identified a novel inter-regulatory loop between ASPP1 and EGR-1. EGR-1-induced ASPP1 is mainly localized at cytoplasm, wherein it elevates Quercetin-induced apoptosis by inhibiting cyto-protective autophagy. These are novel pro-apoptosis mechanisms of ASPP1, independent of the well-documented p53-dependent mechanisms in the nucleus.

## Results

### ASPP1 expression is induced by EGR-1

**Figure 1:**
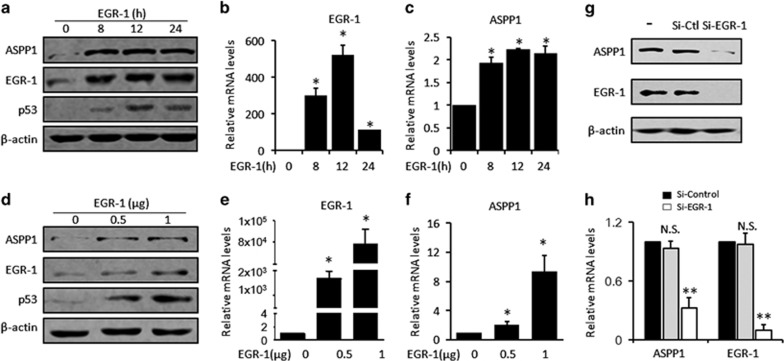
EGR-1 promotes ASPP1 expression at both mRNA and protein levels (**a**–**c**) Protein levels of ASPP1, EGR-1 and p53, a known EGR-1 target, were measured by western blotting (WB) at 0, 8, 12 and 24 h post-transfection with plasmid expressing EGR-1 or empty vector controls. *β*-actin was used as loading controls (**a**). Meanwhile, the mRNA levels of EGR-1 (**b**) and ASPP1 (**c**) were detected by real-time RT-PCR. (**d**–**f**) Protein levels of ASPP1, EGR-1 and p53 were measured by WB analysis after transfection with different doses of EGR-1 plasmids. *β*-actin was used as loading controls (**d**). Meanwhile, the mRNA levels of EGR-1 (**e**) and ASPP1 (**f**) were detected by real-time RT-PCR. (**g** and **h**) ASPP1 and EGR-1 were detected by WB in HCT116 cells both untreated and transfected with Si-EGR-1 or Si-Control. *β*-actin was used as loading controls (**g**). mRNA levels of EGR-1 and ASPP1 were measured by real-time RT-PCR after EGR-1 knockdown (**h**). *P<0.05

To determine whether EGR-1 regulates ASPP1 expression, ASPP1 was examined after forced expression of EGR-1 in HCT116 cells. EGR-1 mRNA and protein were significantly elevated at 8 h and peaked at 12 h post-transfection (Figures 1a and b). Increasing EGR-1 caused a significant induction of p53, a known transcription target of EGR-1, and more interestingly, a remarkable upregulation of ASPP1. Notably, significant accumulation of ASPP1 mRNA and protein occurred also at 8 h post-transfection, and remained in close parallel with the elevated EGR-1 protein throughout the experiments (Figures 1a and c). The expression of ASPP1 was measured also after transfected with different doses of EGR-1. EGR-1 expression and p53 were elevated in a dose-dependent manner (Figures 1d and e). ASPP1 mRNA and protein both exhibited a positive correlation with EGR-1 (Figures 1d and f). These data together suggest that EGR-1 can promote ASPP1 expression.

The effect of EGR-1 on basal expression of ASPP1 was also investigated by using small interfering RNA specifically targeting EGR-1 mRNA (Si-EGR-1). In contrast to the SiRNA controls (Si-control) and untreated control, EGR-1 was remarkably reduced by Si-EGR-1 as revealed by western blotting (WB) and RT-PCR (Figures 1g and h, right panel). ASPP1 expression was simultaneously decreased at both mRNA (about 38%, *P*<0.01) and protein levels upon EGR-1 suppression (Figures 1g and h, left panel), suggesting that EGR-1 is one of crucial regulators in maintaining basal ASPP1 expression.

### Endogenous EGR-1/ASPP1 axis is activated upon various stimulations

**Figure 2:**
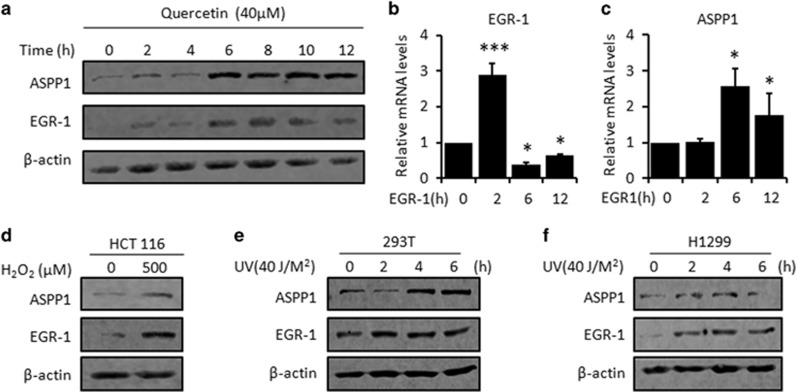
ASPP1 is activated by the endogenous EGR-1 in response to various stimuli. (**a**–**c**) Expression levels of ASPP1 and EGR-1 were determined by WB analysis (**a**) and real-time RT-PCR (**b** and **c**) at different time points after Quercetin treatment in HCT116 cells. (**d**) Expression levels of ASPP1 and EGR-1 were determined by WB analysis in HCT116 cells after exposure to H_2_O_2_ (500 *μ*M) for 4 h. (**e** and **f**) Cells were exposed to UV (40 J/m^n^) for the indicated time periods. Expression levels of ASPP1 and EGR-1 were determined by WB analysis in 293T (**e**) and H1299 (**f**) cells. *β*-actin was used as loading controls for all WB assays. *P<0.05, ***P<0.001

To provide further evidence and to determine the potential stress that could activate endogenous EGR-1/ASPP1 signal, the cells were first treated with anti-oxidative Quercetin, which has been previously reported to activate EGR-1.^33^ Indeed, EGR-1 mRNA was dramatically induced as early as 2 h post treatment and declined to the basal level afterwards, whereas EGR-1 protein level kept rising and turned clearly evident 6–12 h after treatment (Figures 2a and b). The induction of ASPP1 transcription and protein both remained positive correlation with EGR-1 protein (Figures 2a and c). Interestingly, EGR-1 knockdown completely abolished Quercetin-induced ASPP1 expression (Supplementary Figure 1A). These data suggest that endogenous EGR-1 may modulate ASPP1 transcription on Quercetin treatment.

In addition, the activation of EGR-1/ASPP1 is not limited to the Quercetin-treated HCT116 cells. It happened in the cells with different tissue origins and p53 status under various stimulations. For instance, H_2_O_2_ promoted EGR-1/ASPP1 in HCT116 cells (p53 wild type; Figure 2d). A dose-dependent activation of EGR-1/ASPP1 occurred in UV-treated immortalized human embryonic kidney cells, 293T and lung cancer cells, H1299 (p53 null; Figures 2e and f). EGR-1 knockdown abrogated H_2_O_2_ or UV-induced ASPP1 expression (Supplementary Figures 1B and C). Collectively, ASPP1 expression can be regulated by endogenous EGR-1 under unstressed or stressed conditions regardless of p53 status.

### ASPP1 is a novel target gene of EGR-1

**Figure 3:**
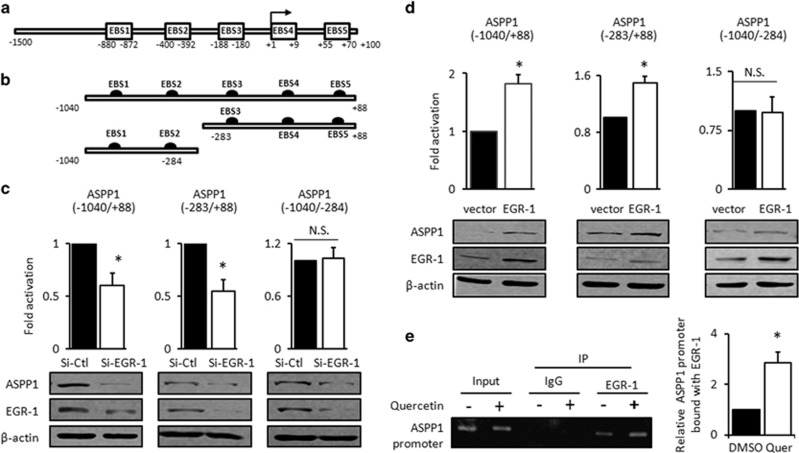
ASPP1 is a transcriptional target of EGR-1 (**a**) ASPP1 promoter region (−1500/+100) contained five consensus EGR-1 binding sites (EBS, EGR-1 binding sites), as predicted by PROMO v3 (http://alggen.lsi.upc.es/cgi-bin/promo_v3/promo/promoinit.cgi?dirDB=TF_8.3) software and shown in rectangle. (**b**) Schematic representation of (−1040/+88) ASPP1 promoter and two mutants, (−1040/−283), containing EBS1-2, or (−1040/−283), containing EBS3-5, were cloned into the luciferase reporter plasmid. (**c** and **d**) Luciferase assay was conducted in with either EGR-1 knockdown (**c**) or EGR-1 overexpression, as described in the 'Materials and Methods' section. Bar graph presented change folds in activation over controls, derived from three independent experiments. S.D. were shown as error bars. (**e**) Cells in the presence or absence of Quercetin were subjected to the ChIP assay. IgG was used as a negative control. The bands were quantified by ImageJ, and normalized with corresponding input. The quantification results derived from three independent experiments were shown in bar graph; *P<0.05

Next, we investigated whether EGR-1 directly regulates ASPP1 expression as a transcription factor. Five potential EGR-1 binding sites (EBS, GNG(T/C/G)GGG(T/C)G)^34^ were identified spanning across 1500 base pair genomic DNA sequence upstream and 100 base pair downstream of transcription start site (TSS) of ASPP1 (Figure 3a). All EBS have also been predicted by PROMO (http://alggen.lsi.upc.es/cgi-bin/promo_v3/promo/promoinit.cgi?dirDB=TF_8.3) bioinformatics tool, suggesting that ASPP1 may be a direct target of EGR-1 by binding with these EBS.

To test the importance of ASPP1 promoter in EGR-1-mediated ASPP1 expression, ASPP1 promoter (−1040/+88), containing all predicted EBS, and two truncated mutants (−1040/−284), containing EBS1-2, and (−283/+88), containing EBS3-5, were cloned into pGL3-basic luciferase reporter plasmid, respectively (Figure 3b). Our results showed that the activities of pGL3-ASPP1-luc and pGL3(−283/+88)-luc were significantly decreased with SiRNA-mediated EGR-1 knockdown by about 50% (*P*<0.05, Figure 3c, left and middle panels). However, EGR-1 failed to affect the luciferase activities of (−1040/−284) reporter under the same conditions (Figure 3c, right panels). Therefore, the predominant binding sites of EGR-1 are located at (−283/+88), which contains predicated EBS3-5. In agreement, the activation of pGL3(−1040/+88) and pGL3(−283/+88) containing luciferase reporter, but not pGL3(−1040/−284) reporter, were promoted by arbitrary EGR-1 expression (*P*<0.05, Figure 3d).

To provide more direct evidence, the binding between EGR-1 and ASPP1 promoter was further examined by a chromatin-immunoprecipitation (ChIP) assay followed by PCR reaction with the pull-down DNA. As shown in Figure 3e, PCR product was only obtained in DNA template precipitated by the anti-EGR-1 antibody, but not in the IgG controls (Figure 3e), suggesting that EGR-1 specifically bound with ASPP1 promoter. Interestingly, this interaction became more pronounced with Quercetin treatment (increased by about 3-folds, *P*<0.05, Figure 3e), which is in support of above data that Quercetin promotes EGR-1 expression and subsequent ASPP1 transcription. Therefore, ASPP1 is a direct target of EGR-1.

### ASPP1 activates EGR-1 in a feedback loop

**Figure 4:**
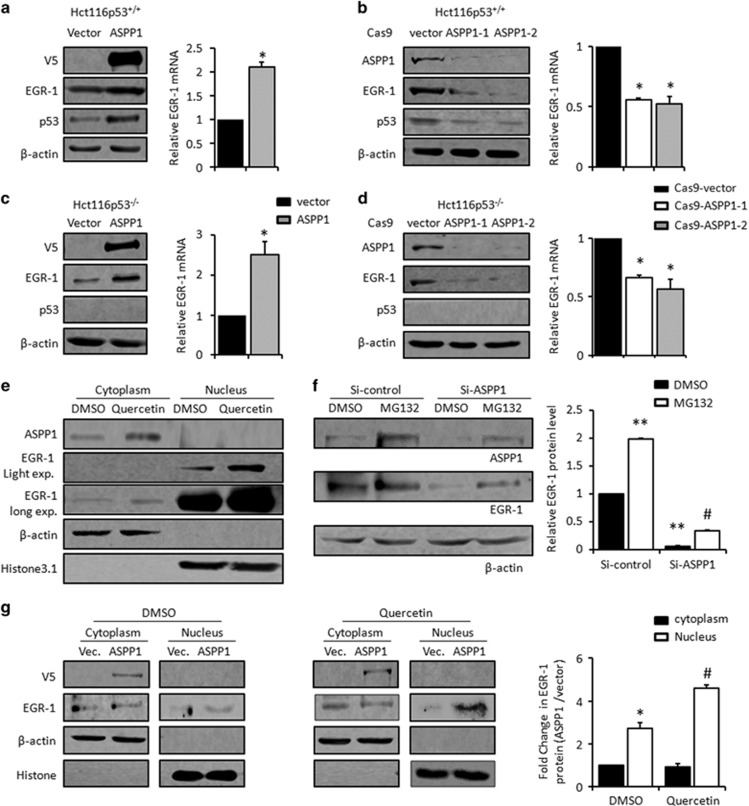
ASPP1 promotes EGR-1 levels in a feedback loop (**a** and **c**) The expression levels of ASPP1 and EGR-1 were analyzed by WB (left) and real-time RT-PCR (right) after being transfected with pcDNA3.1-ASPP1-V5 or empty vector control in HCT116 p53^+/+^ (**a**) or HCT116 p53^−/−^ cells (**c**). (**b** and **d**) The expression levels of ASPP1 and EGR-1 were analyzed by WB (left) and real-time RT-PCR (right) after Crispr/Cas9-mediated ASPP1 silence in HCT116 p53^+/+^ (**b**) or HCT116 p53^−/−^ cells (**d**). (**e**) Cytoplasmic and nuclear fractions were subjected to the WB assay with antibodies specifically targeting ASPP1 or EGR-1 in HCT116 cells in the presence or absence of Quercetin. *β*-Actin and Histone3.1 were used as cytoplasm and nuclear markers, respectively. (**f**) The expression of ASPP1 or EGR-1 were detected by WB after SiRNA-mediated ASPP1 knockdown, with or without MG132 treatment. *β*-Actin was used as loading control. EGR-1 expression level was quantified by ImageJ software. **P<0.01, in comparison with DMSO-treated si-control cells; ^#^*P*<0.05, in comparison with DMSO-treated si-ASPP1 cells. (**g**) The cytoplasmic and nuclear fractions were separated in HCT116 cells transfected with the empty vector control or pcDNA3.1-ASPP1-v5, in the presence or absence of Quercetin. EGR-1 and ASPP1 were detected by WB assay. *β*-Actin and Histone3.1 were used as cytoplasm and nuclear markers, respectively. ImageJ was used to quantify EGR-1 levels. The bar graph showed ASPP1-induced EGR-1 fold changes. *P<0.05, nucleus *versus* cytoplasm; ^#^*P*<0.05, Quercetin *versus* DMSO

EGR-1 can be regulated by its targets, such as p53, in a positive feedback loop. Because ASPP1 is a co-activator of p53, it was next of interest to ask whether ASPP1 regulates EGR-1 in turn. As expected, when exogenous ASPP1 was expressed, EGR-1 mRNA and protein and its target p53, were simultaneously increased (Figure 4a). Conversely, ASPP1 expression was significantly suppressed by the two independent ASPP1-gRNA/Cas9, when compared with control gRNA/Cas9 in HCT116 cells. Interestingly, EGR-1 mRNA and protein levels and its transcription target p53 were all reduced, when ASPP1 expression was disrupted (Figure 4b). Therefore, ASPP1 indeed promotes EGR-1 transcription in a positive feedback loop.

To gain evidence about whether ASPP1 feedback loop is dependent on p53, the same set of experiments was conducted in p53 null HCT116 p53^−/−^, which was originally created by homologous recombination.^35^ Similar to the results obtained in HCT116 p53^+/+^ cells, EGR-1 mRNA and protein were significantly increased with ASPP1 overexpression and reduced with ASPP1 knockdown in HCT116 p53^−/−^ to a similar extent (Figures 4c and d), suggesting that ASPP1 regulates EGR-1 independent of p53. Supportively, the inter-regulatory loop between EGR-1 and ASPP1 was also observed in p53 null H1299 cells (Supplementary Figure 2C). Furthermore, the precision nuclear localization of ASPP1 is perquisite for its activity towards transcriptional activity of p53, however, ASPP1 was predominately localized in the cytoplasm, but not nucleus, no matter Quercetin was added or not, as revealed by a nuclear and cytoplasm fraction assay (Figure 4e). Those data also imply that ASPP1-induced EGR-1 mRNA transcription is an indirect effect independently of p53.

It has also been reported that EGR-1 can be regulated by proteasome-mediated protein degradation in the cytoplasm.^36^ Despite being mainly localized in the nucleus, detectable level of EGR-1 was also found in the cytoplasm (Figure 4e). Proteasome inhibitor MG132 promoted the accumulation of EGR-1, which is in line with previous reports.^36^ Interestingly, MG132 also largely rescued ASPP1 knockdown-induced EGR-1 suppression (Figure 4f), suggesting that ASPP1 regulates EGR-1 mainly via preventing proteasome-mediated protein degradation in the cytoplasm. EGR-1 can transactivate *EGR-1* gene. Therefore, it is possible that ASPP1 promotes EGR-1 transcription by stabilizing EGR-1 protein. In keeping with this notion, ectopic expression of ASPP1 increased the translocation of EGR-1 from the cytoplasm to the nucleus (*P<0.05). This effect was more evident when Quercetin was added (^#^*P*<0.05, Figure 4g). Taken these data together, ASPP1 activates EGR-1 by inhibiting proteasome-mediated EGR-1 degradation and promoting EGR-1 nucleus translocation.

### EGR-1/ASPP1 activation enhances Quercetin-induced apoptosis

**Figure 5:**
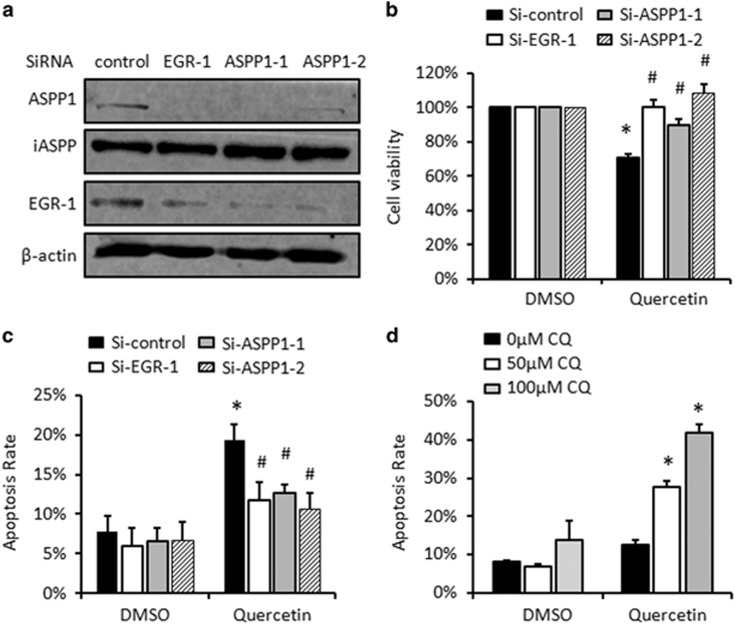
EGR-1/ASPP1 promotes Quercetin-induced apoptosis. (**a**) SiRNA-mediated ASPP1 or EGR-1 knockdown was confirmed by WB. iASPP was a negative control. (**b**) MTT assays were applied to compare the viability of the cells after ASPP1 or EGR-1 knocking down. The values were derived from three independent experiments; S.D. were shown as error bars. (**c**) The cells were treated with Quercetin, stained with Annexin V/PI and subjected to the FACS analysis. The average of apoptotic cells (Annexin V positive) was derived from three independent experiments; S.D. are shown as error bars. *P<0.05, in comparison with untreated control; ^#^*P*<0.05, in comparison with Quercetin-treated Si-control cells. (**d**) Cells were treated with 80 *μ*M Quercetin and 50 *μ*M or 100 *μ*M autophagy inhibitor, chloroquine (CQ) for 24 h. Then they were collected and stained with Annexin V/PI and subjected to the FACS analysis. The average of apoptotic cells (Annexin V positive) was derived from three independent experiments; S.D.are shown as error bars. *P<0.05, in comparison with CQ-untreated control

We further looked at the biological impact generated by EGR-1/ASPP1 activation loop. Quercetin is a promising chemoprevention drug for cancer.^37^ Because EGR-1/ASPP1 was elevated by Quercetin, we first examined the impact of EGR-1/ASPP1 on the anticancer effects of Quercetin by disrupting EGR-1 or ASPP1 expression in HCT116. ASPP1 and EGR-1, but not unrelated ASPP family member iASPP, were inhibited by SiRNA specifically targeting the indicated genes (Figure 5a). As expected, Quercetin treatment resulted in a moderate reduction of cell viability, as revealed by a MTT assay (about 30%, *P*<0.05, Figure 5b). Conversely, knockdown of either EGR-1 or ASPP1 effectively prevented such Quercetin-dependent effect (^#^*P*<0.05, Figure 5b), suggesting that EGR-1/ASPP1 upregulation is critical for Quercetin-mediated toxicity (Figure 5b).

Apoptosis is one of the mechanisms for Quercetin to achieve the anticancer effects.^38^ We, therefore, further examined the apoptotic rate after Quercetin treatment before and after knocking down EGR-1/ASPP1. Quercetin induced about 20% of apoptosis in both HCT116 (*P*<0.05, Figure 5c). Quercetin-induced apoptosis was significantly attenuated in cells transfected with Si-EGR-1 or Si-ASPP1, when compared with those transfected with Si-controls (*P*<0.05, Figure 5c and Supplementary Figure S2A). A similar results were obtained in p53 null H1299 cells (*P*<0.01, Supplementary Figures 2C–E), suggesting that the susceptibility of the cells to Quercetin-induced apoptosis is largely dependent on EGR-1/ASPP1, in a p53-independent manner. It has been supported by mounting evidences that autophagy is a protective mechanism of cell death, treat cells with autophagy inhibitor, chloroquine (CQ), markedly promotes Quercetin-induced apoptosis in a concentration-dependent manner (Figure 5d and Supplementary Figure 2B).

### EGR-1/ASPP1 inhibits cyto-protective autophagy

**Figure 6:**
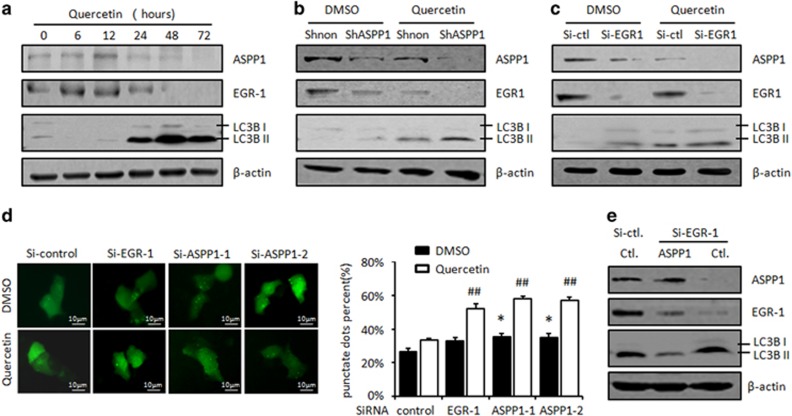
EGR-1/ASPP1 inhibits cyto-protective autophagy. (**a**) Expression levels of ASPP1 and EGR-1 and autophagy protein marker LC3BI/II, were determined by WB analysis in HCT116 cells treated with Quercetin for the different time courses. *β*-Actin was used a loading control. (**b**) The expression of ASPP1, EGR-1 and LC3BI/II were determined by WB analysis in the presence or absence of Quercetin in HCT116/Shnon and HCT116/ShASPP1 stable lines. *β*-Actin was used as a loading control. (**c**) The expression of ASPP1 and LC3BI/II were determined by WB analysis in the presence or absence of Quercetin in HCT116 cells transfected with Si-Control or Si-EGR-1. *β*-Actin was used as a loading control. (**d**) GFP-LC3 puncta (⩾4puncta/cell) containing autophagy cells were counted in Si-ASPP1 or Si-EGR-1 transfected cells, in the presence or absence of Quercetin treatment. *, in comparison with DMSO-treated Si-controls. #, in comparison with Quercetin-treated Si-controls. (**e**) ASPP1 was re-expressed after SiRNA-mediated EGR-1 knockdown. The expression levels of ASPP1, EGR-1 and autophagy protein marker LC3BI/II, were determined by WB. *β*-Actin was used as a loading control

It has been supported by mounting evidence that autophagy can be a protective mechanism of cell death.^39, 40, 41^ Consistent with the previous idea, treating cells with autophagy inhibitor, CQ, Quercetin-induced apoptosis was markedly promoted in a concentration-dependent manner (Figure 5d). As EGR-1/ASPP1 knockdown and autophagy inhibitor generated opposite effects on Quercetin-induced apoptosis, we speculated that EGR-1/ASPP1 signal may promote apoptosis by counteracting with autophagy. In support of this notion, it was found that the common marker for autophagy activity, LC3BII/I, was decreased at 6 and 12 h, and started to increase at 24 h. EGR-1/ASPP1 showed a negative association with the occurrence of autophagy over Quercetin treatment (Figure 6a), further suggesting that EGR-1/ASPP1 may inhibit autophagy.

The impact of ASPP1 on autophagy was then validated by comparing autophagy levels in cells with either overexpression or knockdown of ASPP1. As shown in Figures 6b and c, when ASPP1 and EGR-1 was inhibited by shRNA or SiRNA, specifically targeting either ASPP1 or EGR-1, the indicated proteins were correspondingly decreased, as expected. Interestingly, LC3II/I was also increased with such treatments (Figures 6b and c). The observation was also confirmed in green fluorescent protein (GFP)-tagged LC3 cell models. Autophagy formation was evaluated by calculating the number of cells with GFP-LC3 puncta (⩾4puncta/cell) per GFP-positive cells in the same field (Figure 6d). Autophagy cells were markedly increased in ASPP1 knockdown cells (*P*<0.05, Figure 6d). Moreover, ASPP1 overexpression largely rescued EGR-1 knockdown-induced LC3BII/I accumulation in both HCT116 and H1299 cells (Figure 6e and Supplementary Figure 3A). In addition, the impact of EGR-1 and ASPP1 on autophagy was more pronounced in Quercetin-treated cells as revealed by both WB assay and GFP-LC3 puncta assay (Figures 6b–e and Supplementary Figure 3A).

**Figure 7:**
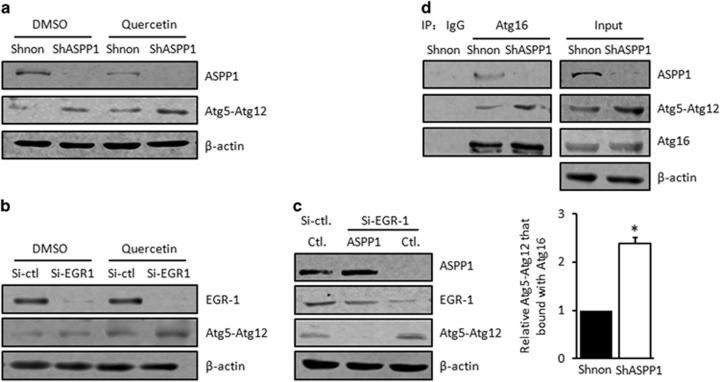
EGR-1/ASPP1 inhibits cyto-protective autophagy by compete Atg5–12 with Atg6 to form a complex. (**a**) The expression of ASPP1, EGR-1 and Atg5–12 were determined by WB analysis in the presence or absence of Quercetin in HCT116/Shnon and HCT116/ShASPP1 stable lines. *β*-Actin was used as a loading control. (**b**) The expression of ASPP1, EGR-1 and Atg5–12 were determined by WB analysis in the presence or absence of Quercetin in HCT116 cells transfected with Si-Control or Si-EGR-1. *β*-Actin was used as a loading control. (**c**) ASPP1 was re-expressed after SiRNA-mediated EGR-1 knockdown. The expression levels of ASPP1, EGR-1 and Atg5–12, were determined by WB. *β*-Actin was used as a loading control. (**d**) Cell lysates derived from HCT116/Shnon and HCT116/ShASPP1 cells were immunoprecipitated with anti-Atg16 antibody. The co-precipitated ASPP1 or Atg5–Atg12 was evaluated by WB. *P<0.05

Furthermore, Atg5–Atg12, a critical complex formed at early stages of autophagy, was induced with ASPP1 and EGR-1 knockdown (Figures 7a and b). As Figure 7c shows, when ASPP1 was rescued in EGR-1 knockdown cells, Atg5–Atg12 was decreased sharply. These results suggest that EGR-1 and ASPP1 inhibit autophagy, possibly by influencing Atg5–Atg12 conjugation. In addition, further complex formation with Atg16 is crucial for the localization of Atg5–Atg12 at autophagosome and subsequent autophagy progress, the interaction between Atg5–Atg12 and Atg16 was further evaluated after ASPP1 knockdown. We found that increased Atg5–Atg12 was presented in Atg16 immunoprecipitate of ASPP1 knockdown HCT116 and H1299 cells, in comparison with the control cells (about twofolds in HCT116 cells, *P*<0.05, Figure 7d and twofolds in H1299 cells, *P*<0.01, Supplementary Figure 3B). Therefore, EGR-1/ASPP1 is one of critical mediators of Quercetin-EGR-1-autophagy signaling, which provides sensible explanations for the pro-apoptotic effect of EGR-1/ASPP1 loop.

## Discussion

Drug resistance remains as a major obstacle to the treatment of cancers. It has been reported that ASPP1 expression levels can influence cell’s response to apoptosis in multiple cancers with different stimulus. However, how ASPP1 expression is regulated is poorly understood. Here, we demonstrate, for the first time, that ASPP1 is a direct target of transcription factor EGR-1. EGR-1 is a crucial regulator not only in maintaining the basal expression of ASPP1 under unstressed conditions, but in rapidly elevating ASPP1 expression in response to stimulations. The data in our study provided evidences that ASPP1 may coordinate EGR-1 activity to determine cell fates, and hence potential targets to improve anticancer efficiency.

**Figure 8:**
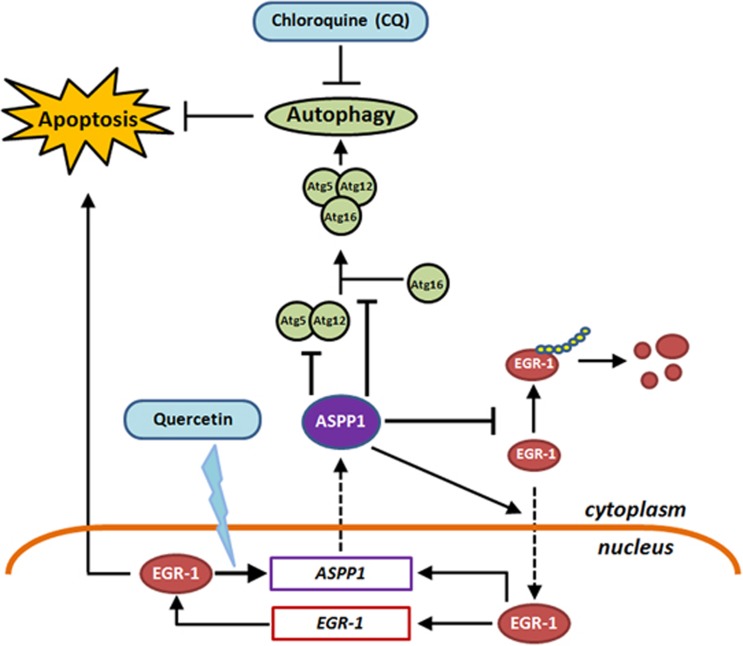
Proposed model of EGR-1/ASPP1 inter-regulatory loop in promoting apoptosis. Upon various stimuli, such as anticancer natural compound Quercetin, EGR-1 is activated, which binds with EBS localized at ASPP1 promoter region and then transactivates ASPP1 expression in the nucleus. Elevated ASPP1 is mainly localized at cytoplasm, which, in turn, inhibits proteasome-mediated EGR-1 degradation and also promotes EGR-1 nuclear import. Activated EGR-1 can further promote apoptosis by transactivating its pro-apoptosis targets, including EGR-1 itself. Meanwhile, ASPP1 binds with Atg5–Atg12 and inhibits their conjugation and further complex formation with Atg16, leading to cyto-protective autophagy inhibition and apoptosis induction

More complicatedly, ASPP1 also promotes EGR-1 mRNA and protein levels in a positive feedback loop. It is known that ASPP1 is a co-activator of p53/73,which is dependent on its direct interaction with p53/p73 and their precise nuclear localization.^42^ Nonetheless, ASPP1 was predominately in the cytoplasm, regardless of Quercetin treatment. Therefore, the alternations of EGR-1 at transcription levels are likely an indirect effect of ASPP1. Interestingly, proteasome inhibitor MG132 largely rescued ASPP1 silencing-induced downregulation of EGR-1, which is in support of previous reports that EGR-1 can be regulated by proteasome-mediated degradation in the cytoplasm.^36^ Moreover, stabilized EGR-1 can transport from the cytoplasm into the nucleus by binding with importin 7 and then fulfill its transcription factor tasks in the nucleus.^43^ In line with this, our present study showed that ASPP1 expression facilitates the nuclear translocation of EGR-1, particularly in Quercetin-treated cells. Hence, although the detailed molecular mechanisms warrant further investigation, these results clearly point to the notion that ASPP1 activates EGR-1 by modulating protein stability and subcellular localization (Figure 8). Notably, EGR-1 promoter contains EBS and EGR-1 protein elevates EGR-1 transcription.^44, 45^ It is thus possible that the ASPP1-induced EGR-1 transcription is due to the activation and stabilization of EGR-1 itself. In addition, p53 and p73 are the transcription targets of EGR-1 and also transactivate EGR-1 in a feedback loop.^34^ It is thus also possible that activated EGR-1 promotes p53/p73, which in turn activates EGR-1 at transcriptional levels, at least in those cells with intact p53/p73 (Figure 8). In support of this notion, p53 levels were parallel with the alteration of EGR-1, when ASPP1 was overexpressed or silenced in HCT116 cells.

In addition, it has been widely recognized that ASPP1 promotes apoptosis via direct interaction with p53 in the nucleus. However, ASPP1 is frequently localized in the cytoplasm, rather than in the nucleus. It remains fascinating how ASPP1 fulfills its suppressor functions in the cytoplasm. Here, we provide important evidence that the cytoplasmic ASPP1 may promote apoptosis via autophagy inhibition. Autophagy represents an evolutionally conserved carbolic process, which breaks down macromolecules or organelles by fusion autophagosome with lysosomes. Typical apoptosis regulators, such as Bcl-2 family members (Bcl-2 and Bcl-xL), Caspase-8 and FADD-like apoptosis regulator (Flip), have been shown to regulate autophagy.^46, 47^ The well-known components of autophagic signal, such as Atg5, beclin1 and Atg4D, can have roles in apoptosis.^48, 49, 50^ It brings up an idea that many proteins may be involved in the cross-talk between autophagy and apoptosis. Here, we found that EGR-1/ASPP1 is an additional factor that has important roles in inhibiting autophagy and promoting apoptosis. Atg5–Atg12 conjugates can form complex with Atg16, which targets Atg5–Atg12 to the autophagic membranes. The formation of Atg5–Atg12/Atg16 is a pivotal initiation step for autophagy. Our data revealed that ASPP1 not only lowered Atg5–Atg12 levels, but also interacted with Atg5–Atg12 and thus disrupted Atg5–Atg12/Atg16 complex forming. Wang *et al.*^51^ demonstrated that another ASPPs member, ASPP2 can inhibit oncogenic Ras-induced autophagy by competing with Atg16 to bind Atg5/Atg12. Interestingly, this activity of ASPP2 is mediated by its N-terminal residues (1–360), which is highly homologous to its family member ASPP1.^9^ It is possible that ASPP1 prevents Atg5–Atg12/Atg16 formation via a similar mechanism as ASPP2 does. It has been reported previously that the third family member iASPP also regulates autophagy by a similar mechanism in keratinocytes.^52^ Considering that iASPP and ASPP1/2 normally produce opposite effects towards cancers, it is possible that ASPP families regulate autophagy in a cell context-dependent manner. It is also noteworthy that the influence of EGR-1 on autophagy can be either positive or negative.^53, 54^ Our data here provide an additional mechanism of pro-autophagic functions of EGR-1 by activating ASPP1. Our results, together with others, suggested that EGR-1 may regulate autophagy in cell context and stimulus-dependent manners.

Furthermore, we cannot exclude the possibility that ASPP1 may promote apoptosis by feedback regulation of EGR-1, either. EGR-1 is an important transcription factor involved in the regulation of multiple pro-apoptosis gene. For example, p53 level is decreased in EGR-1(-/-) cells, which confer to the apoptosis resistance upon ionizing radiation treatment.^34^ EGR-1(-/-) cells also failed to activate another target PTEN and exhibited a resistance phenotype to UV-induced apoptosis.^28^ The transcription activity of EGR-1 has been also found to be critical in c-myc-induced ARF dependent and p53-independent apoptosis.^56^

In summary, a novel EGR-1/ASPP1 inter-regulatory loop has been identified in this study, which provides new molecular insights into the pro-apoptosis functions of cytoplasmic ASPP1 by the stabilization of EGR-1 and the suppression of autophagic initiator Atg5–Atg12/Atg16. Notably, p53 is not required for the newly identified EGR-1/ASPP1 loop. Activating EGR-1/ASPP1 may be a useful strategy to overcome apoptosis resistance by inhibiting cyto-protective autophagy regardless of p53 status.

## Materials and Methods

### Cell culture

Human colorectal carcinoma cells HCT116 p53^+/+^ and HCT116 p53^-/-^, H1299 and renal carcinoma cells CCF-RC-2 were maintained in RPMI-1640 medium (Gibco, Life Technologies, Carlsbad, CA, USA) supplemented with 10% (v/v) FBS (Biological Industries, Beit-Haemek, Israel). 293T cells were cultures in DMEM (Gibco) with 10% (v/v) FBS (Biological Industries). All cells were purchased from the American Type Culture Collection (ATCC) and maintained at 37 °C humidified incubator (Thermo, Waltham, MA, USA) with 5% CO_2_. HCT116/ShASPP1 stable cell line was generated by the infection of lentivirus carrying pLKO.1-shASPP1. Same cells infected with lentivirus pLKO.1 was used a control (HCT116/Shnon).

### RNA interference

RNAi oligos specifically targeting either ASPP1 or EGR-1 was obtained from GenePharma (Suzhou, China). The cells were seeding at approximately 50% confluence before transfection by using Lipofectamine 2000 reagent (Invitrogen, Carlsbad, CA, USA) following the manufacturer’s instruction. Seventy-two hours after transfection, the cells were subjected to a different analysis. The sequence of Si-EGR-1 and Si-ASPP1 were Si-ASPP1-1: 5′-GCACACAGCGCCUUAAAUATT-3′, Si-ASPP1-2: 5′-GAACAAAGGUGUGGCGUAUTT-3′ and Si-EGR-1: 5′-GGCAUACCAAGAUCCACUUTT-3′.

### Construction of Cas9 system plasmid

The modified px458 plasmid contains two gRNA, cas9-GFP and puromycin selection elements. Knocking down exon2 and exon3 in ASPP1 leads to ASPP1 sliencing according to pervious reported mouse model.^57^ The same region was therefore designed to be targeted by Cas9 system. gRNA was designed using http://www.broadinstitute.org/rnai/public/analysis-tools/sgrna-design. The sequence of these oligonucleotides are as follows: Cas9 ASPP1-1 forward 5′-gagaatggcatgaacccggg-3′, downstream 5′-TATAATCCCAGAACTCTGGG-3′ Cas9 ASPP1-2 forward 5′-GGTATCTCAAAAATCAAGGA-3′, downstream 5′-tctagccacttgtaagtgca-3′. The standard dimer formation protocol was used to make the oligonucleotides become dimers. BbsI was first used to subclone dimer into one of the insert site of the modified px458 vector. After sequencing to make sure that the dimer has been inserted successfully, another enzyme called BSAI was used to make the second dimers inserted into the plasmid. The plasmids were transfected into cells by Lipofectamine 2000 reagent (Invitrogen) for indicated experiments.

### Luciferase reporter assay

The ASPP1 promoter (−1040/+88) was amplified from HEK293 cells and cloned into pGL3-Basic (pGL3-ASPP1). The truncated fragments, pGL3-ASPP1(−1040/−284) and pGL3-ASPP1(−283/+88) were produced by the same method by using pGL3-ASPP1 (−1040/+88) as a template. All the constructs were sequenced and verified. The cells were cotransfected with 500 ng reporter constructs, 300 ng plasmids or 20 pmol siRNA and 7 ng renilla. The cells were lysed 24 h after transfection and luciferase activities were assessed using Dual-Luciferases Reporter Assay System (Promega, Madison, WI, USA). The luciferase activities were measured in Fluorescence microplate reader at absorbance of 528 nm for luciferase and 405 nm for renilla. The relative luciferase activities were calculated by the ratio of lucicerase/renilla. The relative luciferase activities in the controls were normalized to 1.

### Western blot

Total protein lysates were obtained from cells in UREA buffer (8 M urea, 1 M thiourea, 0.5% CHAPS, 50 mM DTT and 24 mM Spermine). Protein concentrations were measured by Bradford method. Same amount proteins were separated by SDS-PAGE and transferred to PVDF membranes (Millipore, Billerica, MA, USA), followed by blocking with 5% skimmed milk for 1 h at room temperature. Primary antibodies used were listed as bellow: ASPP1 (Sigma, St. Louis, MO, USA), iASPP (Sigma), EGR-1 (Cell Signaling Technology, Danvers, MA, USA), DO-1 (Abcam, Cambridge, MA, USA), LC3B (Cell Signaling Technology), Beclin1 (Abcam), V5 (Trevigen, Gaithersburg, MD, USA) and *β*-actin (Sungene, Tianjin, China). The immunoblots were incubated with the indicated primary antibodies overnight at 4 °C and then horseradish peroxidase (HRP)-conjugated anti-rabbit or anti-mouse secondary antibodies (Abcam) at room temperature for 1 h. The signals were visualized by ECL. The membrane was then ready for scanning by Image studio system.

### RNA extraction and quantitative RT-PCR

Total RNA was extracted using Trizol Reagent (Invitrogen) according to the manufacturer’s instructions. Total 2 *μ*g RNA was used for cDNA synthesize by using TaqMan Reverse Transcription (RT) Reagents Kit (Applied Biosystems, Branchburg, NJ, USA). After the RT reaction, the cDNA was diluted by 10-fold and subjected to the analysis of quantitative RT-PCR. Quantitative real-time PCR was carried out by using SYBR Premix Ex Taq kit II (Takara, Dalian, China) on the Vii7 real-time PCR machine (Applied Biosystems, Branchburg, NJ, USA). The conditions used were as follows: 95 °C for 30 s (s), 40 cycles of 95 °C for 5 s, 58 °C for 34 s and last stage at 95 °C for 15 s, 60 °C for 1 min (min), 95 °C for 15 s. mRNA levels were calculated according to the cycle threshold (*Ct*) value. The housekeeping gene GAPDH was used as an internal control. The primer sequences used in this study are as listed below. GAPDH forward: 5′-CGACCACTTTGTCAAGCTCA-3′, reverse: 5′-ACTGAGTGTGGCAGGGACTC-3′ EGR-1 forward: 5′-AGCACCTGACCGCAGAGTCTT-3′, reverse: 5′-CACTAGGCCACTGACCAAGCT-3′ ASPP1 forward: 5′-GCCAAGGAACAGCGTTTACA-3′, reverse: 5′-GCAGACAGATTGCCGTTCAT-3′.

### Chromatin immunoprecipitation*assay*

Briefly, asynchronously growing HCT116 cells were incubated with formaldehyde to yield protein–DNA cross-link complexes. The cross-linked chromatin was then purified, diluted with lysis buffer at 1:5 and sheared by sonication. After preclearing with protein G-agarose beads (GE Healthcare, Uppsala, Sweden), the chromatin was divided equally into two groups for further immunoprecipitation reaction with either anti-EGR-1, and nonspecific immunoglobulin G (IgG) derived from same species. The immunoprecipitates were pelleted by centrifugation and then incubated at 65 °C to reverse the protein–DNA cross-linking. The DNA was extracted by the Qiagen PCR product purification kit and subjected to PCR reaction with primers list as follows: ChIP-ASPP1-forward: 5′-CGGGAAGCCCCGCCCCTCTCC-3′, reverse: 5′-CAGCCCCAGCCCGACAGCCTGC-3′.

### MTT assay

HCT116 cell were seeded into 24-well plates at an appropriate density for RNAi transfection. The cells were reseeded in 96-well culture plates 48 h after transfection and treated with Quercetin for additional 24 h. Subsequently, the cell viabilities were evaluated by MTT assays. MTT (Sigma) stock solution diluted to 0.5 mg/ml with PBS was added to each well and incubated at 37 °C for 4 h. After that, DMSO was added to dissolve the formazan after carefully aspirated MTT. Optical density was measured with a spectrometer at 490 nm.

### Apoptosis assays

The cells were seeded into 24-well plates, and treated with DMSO or Quercetin for 24 h as indicated in the figures. Both suspended and attached cells were collected gently after the treatment. A total 1 × 10^5^ cells were thoroughly mixed with 5 *μ*l Annexin V/FITC, allowing reaction in the dark for 10 min and then stained with 5 *μ*l propidium iodide (PI) solution for 5 min at room temperature. The total volume was adjusted to 500 *μ*l by adding 1 × PBS and the rate of apoptosis was measured by FACS within 1 h.

### Cell fraction

About 3 × 10^6^ cells were washed with PBS and resuspended in 200 *μ*l of cytoplasm lysis buffer A (10 mM HEPES, pH 7.9, 10 mM KCl, 1.5 mM MgCl_2_, 0.5 mM mercaptoethanol and protease inhibitor). After vortexing for 15 s and incubation for 15–20 min on ice, 5 *μ*l 10% NP-40 was added into the mixture, followed by another round of vortex and incubation. The cytoplasm fraction was then obtained by collecting the supernatant after centrifuging at 16 000 r.p.m. for 15 min at 4 °C. The pellet was washed three times by buffer A. The resulting pellet was resuspended in three volume nuclear lysis buffer B (20 mM HEPES, pH 7.6, 20% glycerol, 50 mM NaCl, 1.5 mM MgCl_2_, 0.1% NP-40 and 5 mM DTT), followed by sonication and vortex. The nuclear fraction was obtained by centrifuge at 16 000 r.p.m. for 30 min at 4 °C.

### Measurement of autophagosome formation

The cells were transfected with pEGFP-LC3 plasmid using Lipofectamine 2000 reagent (Invitrogen) according to the manufacturer’s protocol. Stable lines were selected by treating cells with G418 for about 3 weeks. GFP-LC3 puncta were photographed using a fluorescence microscopy (Olympus, Hamburg, Germany) in cells with or without Quercetin treatment. The cells with more than four GFP-LC3 puncta were counted under blinded conditions. A minimum of 200 total cells were counted at different random fields of each treatment.

### Statistical analysis

Data were expressed as the mean±S.D. Statistical analysis was performed using Student’s *t*-test, and *P*<0.05 was considered significant.
