# Supplementary material for: EGR-1/ASPP1 inter-regulatory loop promotes apoptosis by inhibiting cyto-protective autophagy
Source: Cell Death Dis. 2017 Jun 8;8(6):e2869–. doi: 10.1038/cddis.2017.268 (PMC5520923; doi:10.1038/cddis.2017.268)
Supplement: Supplementary Information [file cddis2017268x1.docx]

**Supplementary Materials and Methods**

***Cell culture***

H1299/ShASPP1 stable cell line was generated by the infection of lentivirus carrying pLKO.1-shASPP1. Same cells infected with lentivirus pLKO.1-vector was used a control (H1299/Shnon).

**Supplementary Figure Legends**

**Figure S1. related to Figure 2.** **Various stimulations induce ASPP1 expression in a EGR-1 dependent manner.** (A-B) Expression levels of ASPP1 and EGR-1 were determined by WB after exposure to H_2_O_2_ (500μM) for 4h (A) or Quercetin (80μM) for 24h (B) in the control and EGR-1 knockdown HCT116 cells. (C) Expression levels of ASPP1 and EGR-1 were determined by WB 4hours after exposed to UV (40J/m^2^) in the control and EGR-1 knockdown 293T cells. β-actin was used as a loading control.

**Figure S2. related to Figure 5.** **EGR-1/ASPP1 activation enhances Quercetin-induced apoptosis.** (A) The representative images showed Quercetin-induced apoptosis in HCT116 cells with or without EGR-1 or ASPP1 knockdown. (B) The representative images showed Quercetin-induced apoptosis in HCT116 cells with or without 50μM or 100μM Chloroquine (CQ), an autophagy inhibitor, for 24h. (C) SiRNA-mediated ASPP1 or EGR-1 knockdown was confirmed by WB in H1299. iASPP was used as a negative control. β-actin was used as a loading control. (D-E) The above H1299 cells were subjected to 80μM Quercetin treatment for 24h. Apoptosis was determined by Annexin V/PI staining and analyzed by FACS. The representative images of apoptosis assay results were shown in (D) and the average of apoptosis rates (Annexin V positive) derived from three independent experiments was calculated and plotted in (E). S.D. are shown as error bars. *, *P*<0.05, in comparison with untreated control; #, *P*<0.05, in comparison with Quercetin treated Si-control cells.

**Figure S3. related to Figure 6. EGR-1/ASPP1 inhibits cyto-protective autophagy** (A) ASPP1 was re-expressed after SiRNA-mediated EGR-1 knockdown. The expression levels of ASPP1, EGR-1 and autophagy marker LC3BI/II, were determined by WB. (B) Cell lysates derived from H1299/Shnon and H1299/ShASPP1 cells were immunoprecipitated with anti-Atg16 antibody. The co-precipitated ASPP1 or Atg5-Atg12 was evaluated by WB. **, *P*<0.01.
