# Supplementary figures and images for: EGR-1/ASPP1 inter-regulatory loop promotes apoptosis by inhibiting cyto-protective autophagy
Source: Cell Death Dis. 2017 Jun 8;8(6):e2869–. doi: 10.1038/cddis.2017.268 (PMC5520923; doi:10.1038/cddis.2017.268)

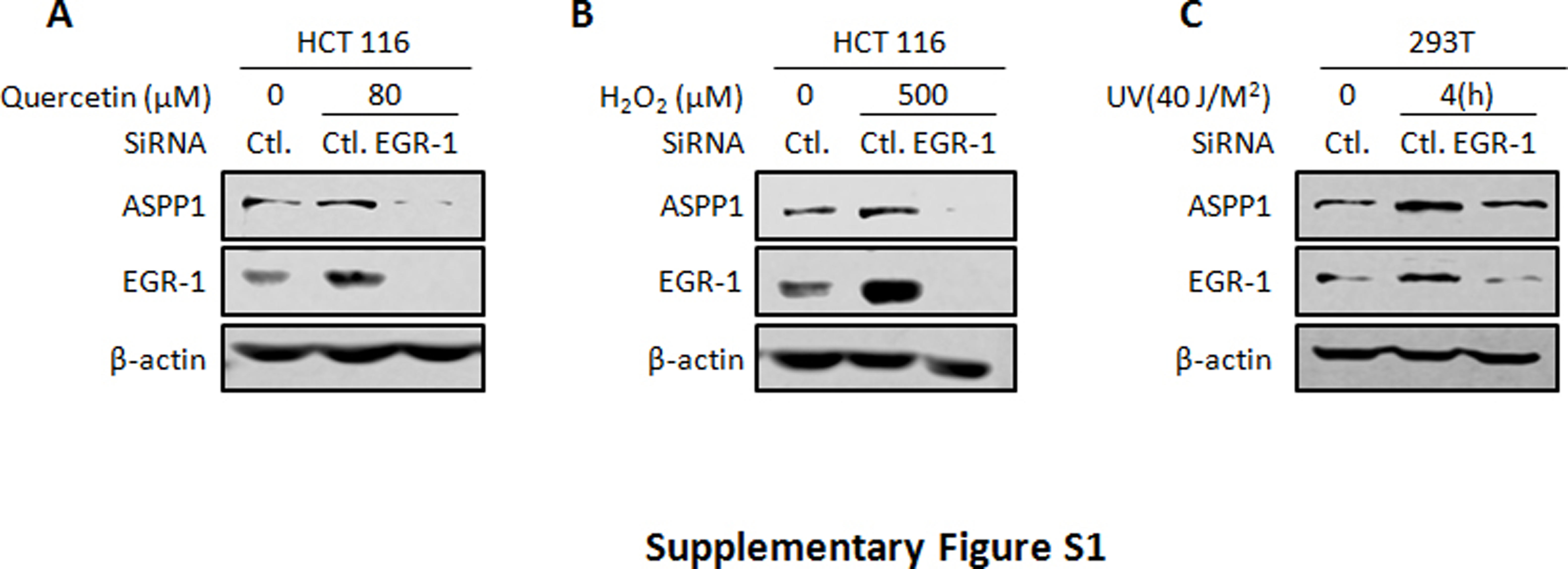

Supplement: Supplementary Figure 1 [file cddis2017268x2.tif]

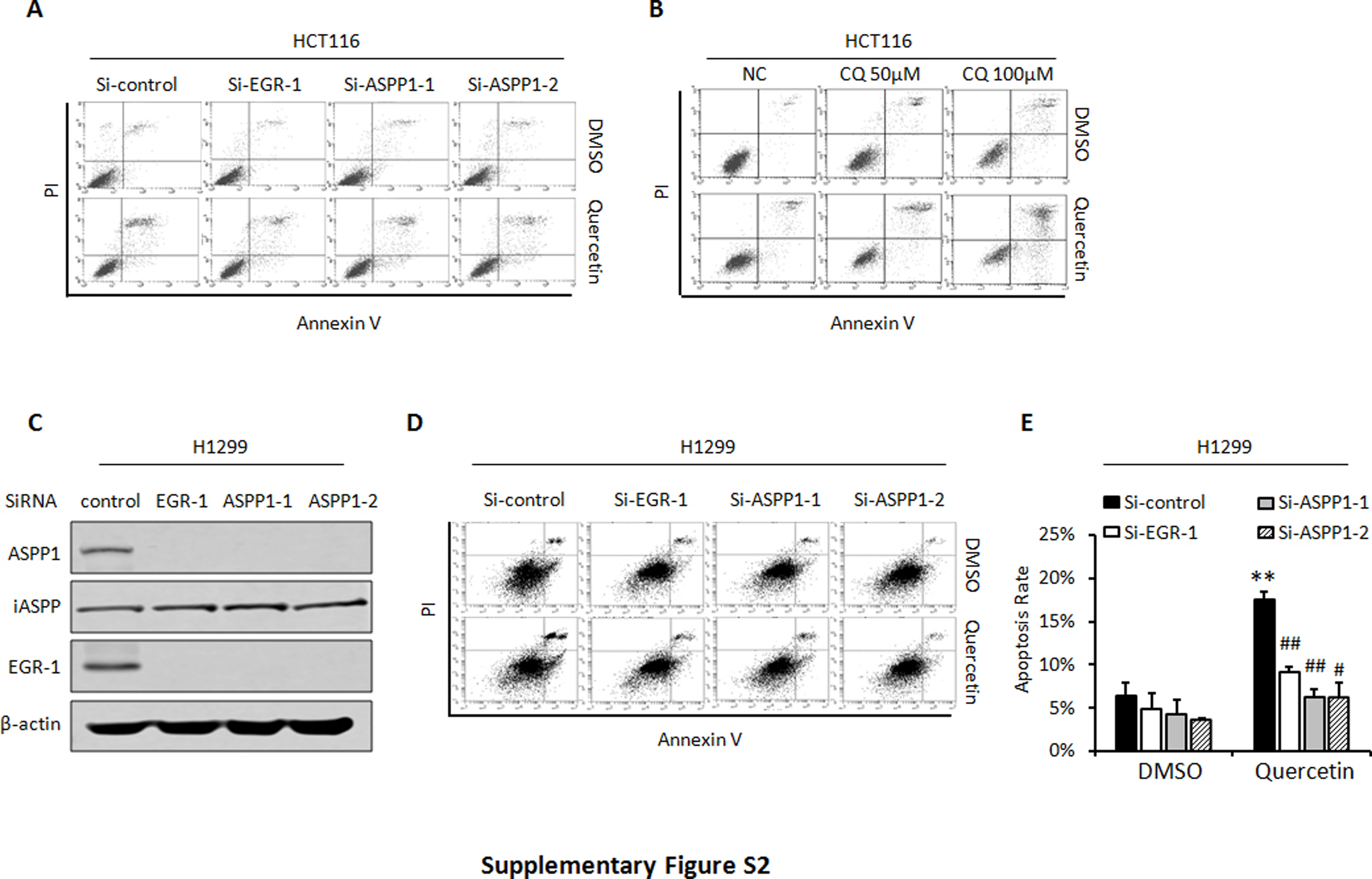

Supplement: Supplementary Figure 2 [file cddis2017268x3.tif]

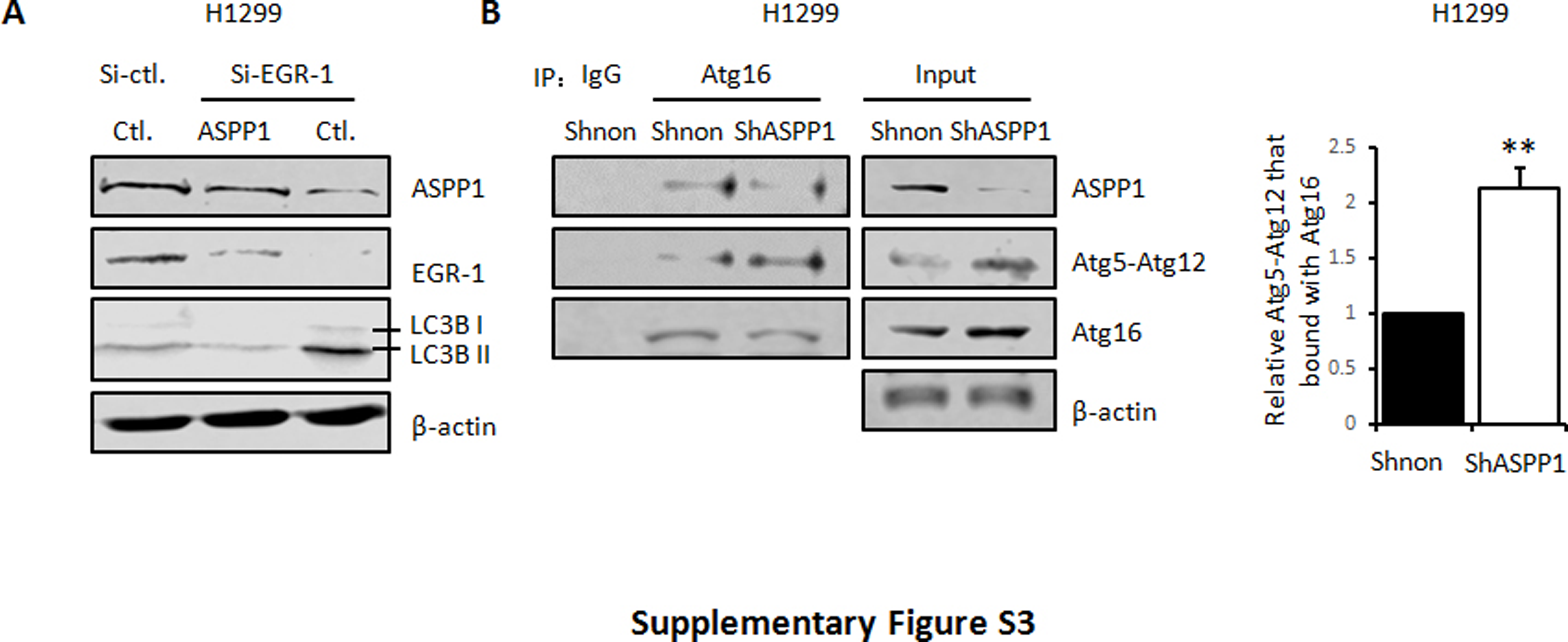

Supplement: Supplementary Figure 3 [file cddis2017268x4.tif]
